# Malignant melanoma of the lung: a case report

**Published:** 2012-04-14

**Authors:** Aziz Ouarssani, Fouad Atoini, Rafik Reda, Fatima Ait Lhou, Mustapha Idrissi Rguibi

**Affiliations:** 1 Military Hospital Moulay Ismail, Meknes, Morroco

**Keywords:** Primary lung tumor, malignant melanoma, metastasis

## Abstract

Primary melanoma of the lung is an extremely rare pathological entity and sparsely reported in the literature. A 68-year-old man was admitted with 3 months history of cough, sputum production, dyspnea, hemoptysis and chest pain. The chest radiography demonstrated bilateral mass lesion and thoracal computerized (CT) showed a bilateral tissu mass with left parietal invasion. Bronchoscopy revealed a large polypoidal tumor arising from the left lower lobe bronchus, histology at bronchial biopsy revealed a malignant melanoma. Surgical biopsy of the left parietal mass was confirmed by invasive malignant melanoma. Primary melanoma of the lung represents a rare pathological entity; careful interpretation of histopathological information in correlation with all other findings from clinical studies can establish a diagnosis.

## Introduction

Melanoma is widely known as the most lethal of all skin cancers, and pulmonary metastases are the most common presentation of advanced disease [1]. Primary pulmonary melanoma is the rarest type of visceral melanoma: more than 30 cases have been reported in the literature [2]. We describe the case of a patient in whom a primary melanoma of the left lower lobe bronchus was diagnosed, and we discuss the cause and outcome of this rare pathology.

### Patient and observation

**Figure 1:**
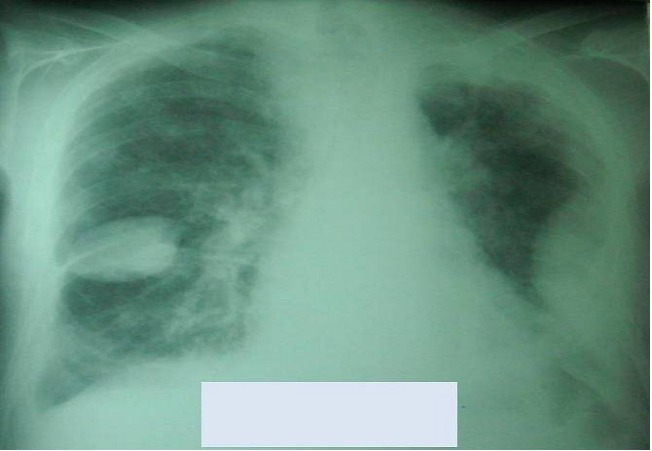
The chest radiography demonstrated bilateral mass lesion

**Figure 2:**
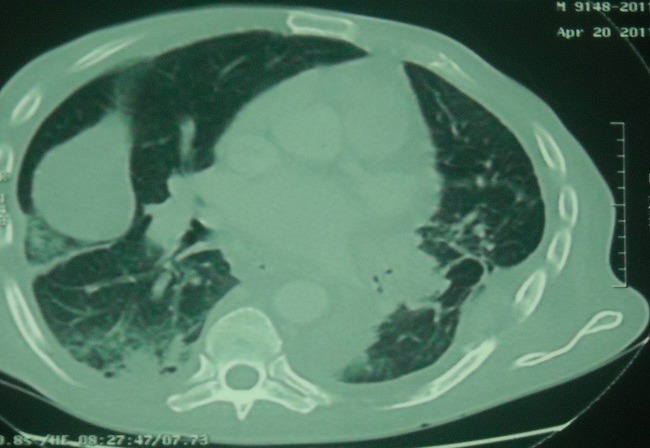
Thoracal CT showed mass lesion in left lung with parietal invasion

**Figure 3:**
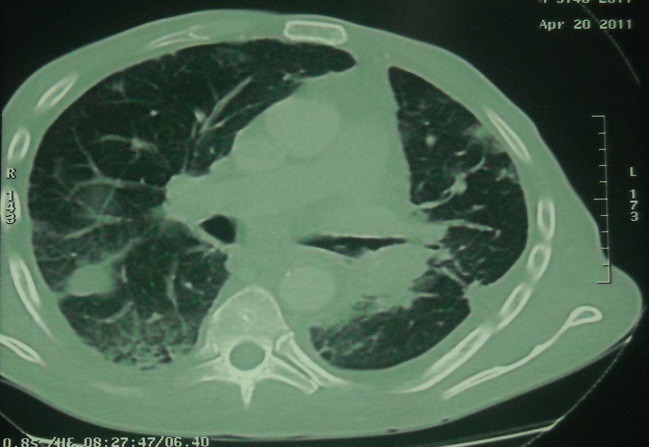
Thoracal CT showed mass lesion in right lower lobe

A 68-year-old man was admitted with a 3 months history of cough, sputum production, dyspnea, hemoptysis, chest pain, and weight loss. Physical examination found a patient cachectic with a mass of right chest wall pain on palpation. The chest radiography demonstrated bilateral mass lesion (Figure 1) and thoracal CT showed mass lesion in left lung with parietal invasion (Figure 2) and in right lower lobe (Figure 3). Complete blood count showed anemia (Hb:9.7mg/dl). Erythrocyte sedimentation rate was 94 mm/hr. Bronchoscopic examination revealed a large polypoidal tumor arising from the left lower lobe bronchus, histology at bronchial biopsy revealed a malignant melanoma :the cytoplasm of the tumor cells contained granular, brown pigment compatible with melanin, the tumor extended to the bronchial epithelium. Immunohistochemical stains were strongly positive for antibodies to S-100 protein and humain melanoma black-45 which confirmed the diagnosis. Surgical biopsy of the left parietal mass was confirmed by invasive malignant melanoma.

To exclude the possibility of metastasis from occult primary malignant melanoma, an extensive examination was carried out, the patient had no past history of skin tumor, and we could not find any skin, external ear, or ocular lesions. Gastrointestinal endoscopy, colonoscopy, and endoscopy of the nasal cavity were performed, and no possible primary tumor was detected. The final diagnosis was primary melanoma of the lung, the patient was given chemotherapy consisting of the docarbazinze, nimustine hydrochloride and vincristine, the patient died of tumor progression one month after first cycle of chemotherapy.

## Discussion

Primary malignant melanoma of the lung is a very rare neoplasm; accounting for 0, 01% of all lung tumours. It is frequently endobronchial and manifest with symptoms of cough, hemoptysis, postobstructive pneumonia, or atelectasie. In 30% of the cases, primary malignant melanoma of the lung is an incidental finding on chest radiography [3, 4]. The proposed criteria for diagnosis include the following criteria of Jensen [5, 6] : 1) Junctional changes like «dropping off» or «nesting » of melanoma cells just beneath the bronchial epithelium; 2) Invasion of the bronchial epithelium by melanoma cells; 3) Malignant melanoma associated with these epithelial changes; 4) A solitary lung tumour; 5) No history of a cutaneous, mucous membrane or ocular melanoma; 6) Absence of any other detectable tumour at the time of diagnosis

These criteria should be revised with the advent of whole-body PET scan: in case of a single pulmonary uptake with histological confirmation of lung lesion, this association would increase the likelihood of diagnosis of primary malignant melanoma of the lung. The present case fulfils the aforementioned diagnostic criteria.

Why should malignant melanoma develop in the bronchi when melanocytes are not apparently present in the normal respiratory tract? Normally, melanocytes migrate to the epidermis and the dermoepidermal junction of the skin, but they may migrate to the visceral during embryogenesis. this has been suggested for the oesophagus and the larynx and may be the case in lung too [7].

The main differential diagnosis is melanocytic carcinoid tumour, melanotic paraganglioma, melanotic schwanoma and pulmonary metastasis of a malignant melanoma. Treatment of choice is surgical resection of the tumour with an oncologically adequate margin, the role of postoperatively adjuvant chemotherapy or radiotherapy either singly or in combination is not known, adjuvant interferon ? was also received on postoperatively [8].

In most cases, patients with primary malignant melanoma of the lung had a poor prognosis, in some reports, the surgical approach with adjuvant chemotherapy /immunochemotherapy provided long-term survival [9]. Our patient died one month after first cycle of chemotherapy.

## Conclusion

Primary malignant melanoma of the lung is exceptional: the diagnosis is based on old criteria, the advent of the PET scan will definitely change the attitude diagnosis but the prognosis is poor.
